# Sarco(endo)plasmic reticulum Ca^2+^-ATPase function is impaired in skeletal and cardiac muscles from young DBA/2J mdx mice

**DOI:** 10.1016/j.isci.2022.104972

**Published:** 2022-08-18

**Authors:** Riley E.G. Cleverdon, Jessica L. Braun, Mia S. Geromella, Kennedy C. Whitley, Daniel M. Marko, Sophie I. Hamstra, Brian D. Roy, Rebecca E.K. MacPherson, Val A. Fajardo

**Affiliations:** 1 Department of Kinesiology, Brock University, 1812 Sir Isaac Brock Way, St. Catharines, ON, L2S 3A1, Canada; 2 Centre for Bone and Muscle Health, Brock University, St. Catharines, ON, Canada; 3 Department of Health Sciences, Brock University, St. Catharines, ON L2S 3A1, Canada; 4 Centre for Neuroscience, Brock University, St. Catharines, ON L2S 3A1, Canada

**Keywords:** Pathophysiology, cell biology

## Abstract

The DBA/2J (D2) *mdx* mouse is a more severe model of Duchenne muscular dystrophy when compared to the traditional C57BL/10 (C57) *mdx* mouse. Here, we questioned whether sarco(endo)plasmic reticulum Ca^2+^-ATPase (SERCA) function would differ in muscles from young D2 and C57 *mdx* mice. Both D2 and C57 *mdx* mice exhibited signs of impaired Ca^2+^ uptake in the gastrocnemius, diaphragm, and left ventricle; however, the level of impairment was more severe in D2 *mdx* mice. Reductions in maximal SERCA activity were also more prominent in the D2 *mdx* gastrocnemius and diaphragm when compared to those from C57 *mdx* mice; however, there were no differences detected in the left ventricle. Across all muscles, D2 *mdx* mice had the highest levels of oxidative stress as indicated by protein nitrosylation and/or nitration. In conclusion, our study shows that SERCA function is more impaired in young D2 *mdx* mice compared with age-matched C57 *mdx* mice.

## Highlights

• Ca^2+^ uptake is severely impaired in muscles from young DBA/2J (D2) *mdx* mice
• Maximal SERCA activity is lowered to a greater degree in muscles from D2 *mdx* mice
• Muscles from young D2 *mdx* mice have higher levels of oxidative/nitrosative stress
• Worsened SERCA function may contribute to worsened muscle pathology in D2 *mdx* mice

## Graphical abstract

**Figure:**
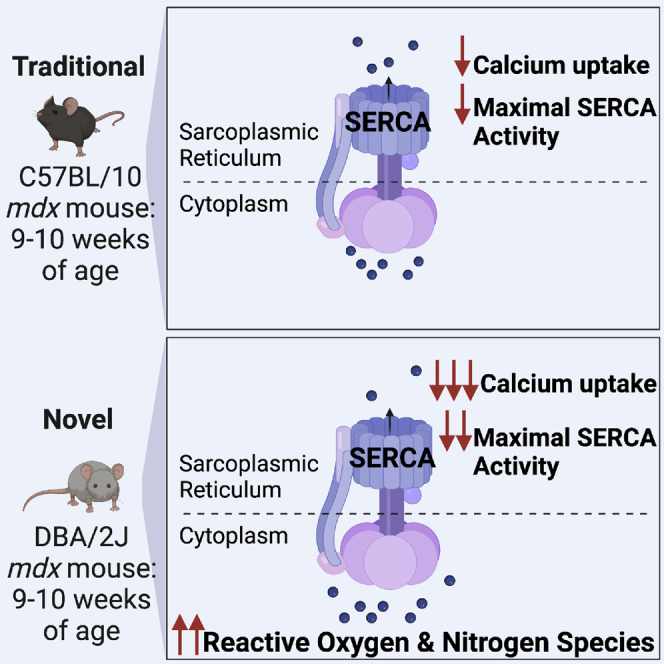

## Introduction

Duchenne muscular dystrophy (DMD) is an X-linked recessive muscle wasting disease, affecting approximately 1 in 5,000 boys worldwide (Mah et al., 2014; Mendell et al., 2012). Symptoms of DMD typically begin at three or four years of age and with no cure to date, patients usually succumb to cardiorespiratory complications from the disease by the third or fourth decade of life (Emery, 2002; Mavrogeni et al., 2015; Straub et al., 2016). DMD is caused by the complete loss of dystrophin, a protein that normally connects the cytoskeleton and extracellular matrix to the sarcolemma thereby leading to excessive membrane tearing and myofiber damage/degeneration (Blake et al., 2002). Loss of dystrophin is the initial insult of DMD; however, there are several secondary consequences of the disease including chronic inflammation, oxidative stress, and calcium (Ca^2+^) overload (Tubridy et al., 2001). Combined, these processes ultimately lead to the pathological hallmarks of DMD which include muscle degeneration, muscle necrosis, and fatty and fibrotic deposition (Shin et al., 2013).

Ca^2+^ is a potent signaling molecule largely responsible for excitation-contraction coupling (ECC) in skeletal and cardiac muscle. Two major Ca^2+^ regulatory proteins involved in muscle ECC are the ryanodine receptor (RyR) and sarco(endo)plasmic reticulum Ca^2+^-ATPase (SERCA) pump, located in the sarcoplasmic reticulum (SR). RyR is responsible for the release of Ca^2+^ from the SR into the myoplasm, which initiates cross-bridge formation and force generation, whereas SERCA catalyzes the reuptake of Ca^2+^ into the SR to initiate muscle relaxation (Santulli et al., 2018; Tupling, 2004, 2009). Several studies have shown that if SR Ca^2+^ handling is dysregulated, Ca^2+^ can become chronically elevated in the myoplasm, leading to muscle damage and weakness. In fact, impaired Ca^2+^ handling is a major part of the pathology in the muscles of patients living with DMD, whereby Ca^2+^ overload exacerbates oxidative stress, inflammation, and cellular necrosis (Robert et al., 2001; Turner et al., 1988). SERCA specifically has been implicated in dystrophic pathology, and several studies have shown that SERCA is impaired in skeletal and cardiac muscles from the murine C57BL/10ScSn (C57) *mdx* model of DMD (Gehrig et al., 2012; Schneider et al., 2013; Voit et al., 2017). This is in part owing to elevations in reactive oxygen and nitrogen species (RONS) in *mdx* muscle that can damage the SERCA pumps (Gehrig et al., 2012). The SERCA pumps contain several residues that are susceptible to RONS-mediated modifications such as nitrosylation and nitration, ultimately impairing their ability to transport Ca^2+^ (Viner et al., 1996, 1997, 1999a, 1999b).

The C57 *mdx* mouse, originally discovered in 1984 (Bulfield et al., 1984), has served as the dominant *in vivo* model to study cellular mechanisms and potential therapies for DMD. However, with a mild dystrophic pathology that is slow to progress, researchers have sought out alternative models that may better recapitulate human DMD (Swiderski and Lynch, 2021). For example, previous research has shown that SERCA pump function is more severely impaired in the *mdx*/utrophin double knockout (dko) mouse (Schneider et al., 2013; Voit et al., 2017)—a severe DMD mouse model where both dystrophin and its functional homolog utrophin are completely absent (Deconinck et al., 1997; Grady et al., 1997). This suggests that SERCA dysfunction progresses along with disease severity, where more severe models of DMD will display more severe impairments to SERCA function.

Recently, the C57 *mdx* mouse was backcrossed onto a DBA/2J (D2) background, and unlike its predecessor, the D2 *mdx* mouse presents with early onset DMD pathology that is worsened with pronounced muscle weakness and damage, degeneration, fibrosis, necrosis, and inflammation (Coley et al., 2016; Hammers et al., 2020; van Putten et al., 2019). The underlying mechanisms behind the worsened pathology displayed in D2 *mdx* mice compared to C57 *mdx* mice are still under investigation, though a recent report has implicated elevated TGF-β signaling (Mázala et al., 2020). However, to our knowledge SERCA function has yet to be characterized in the D2 *mdx* mouse. Therefore, we examined SERCA function in 8-10-week-old D2 and C57 *mdx* mice. This cohort was selected based on previous studies showing that D2 *mdx* mice at this age display muscle weakness and pathology, whereas the C57 *mdx* mice do not (Coley et al., 2016; Hammers et al., 2020; van Putten et al., 2019). We hypothesized that impairments to SERCA function would progress according to disease severity, where skeletal and cardiac muscles from D2 *mdx* mice would display greater impairments to SERCA function than C57 *mdx* mice.

## Results

### Muscle weakness and wasting in the C57 mdx versus D2 mdx mice

**Figure 1:**
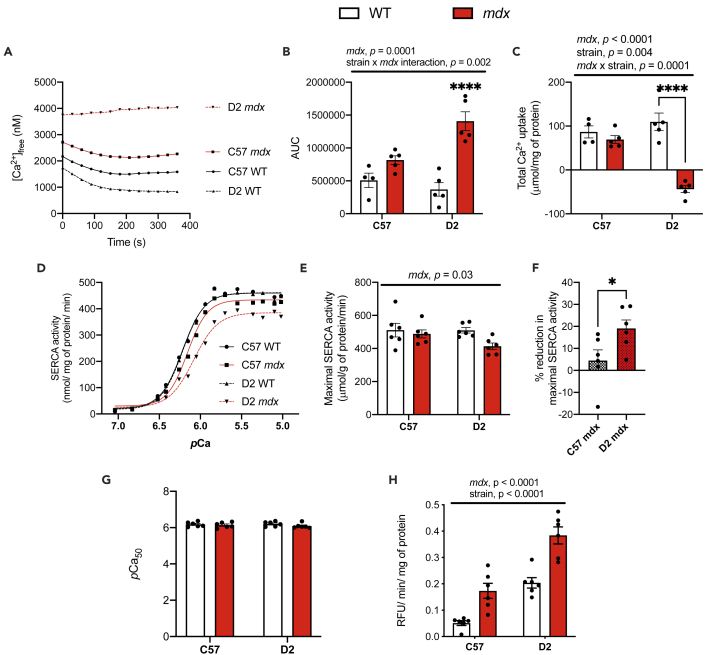
Impairments in Ca^2+^ uptake and maximal SERCA activity in gastrocnemius muscles from *mdx* mice are more prominent on the D2 background strain (A) Ca^2+^ uptake curves from gastrocnemius homogenates. (B) Area under the curve (AUC) of Ca^2+^ uptake curves. (C) Total Ca^2+^ uptake normalized to mg of protein loaded. (D) Ca^2+^-dependent SERCA activity curves in D2 and C57 -WT and -*mdx* gastrocnemius muscles. (E) Maximal SERCA activity expressed as μmol/g of protein/min. (F) The % reduction in maximal SERCA activity in C57- and D2 *mdx* mice. (G) *p*Ca_50_ of SERCA activity. (H) Calpain activity in gastrocnemius muscles from D2 and C57 -WT and -*mdx* gastrocnemius muscles expressed as RFU min/mg of protein. Two-way ANOVAs were performed with a Sidak’s multiple comparisons test, ∗p < 0.05, ∗∗p < 0.01, ∗∗∗p < 0.001, and ∗∗∗∗p < 0.0001 n = 4-6. Main effects and interactions expressed in bars over the graph. Values are represented as mean ± SEM. For F, a Student’s t-test was used, ∗p < 0.05.

We first sought to verify the phenotypical weakness and atrophy previously reported in the D2 *mdx* mice. Compared with their respective WT controls, serum creatine kinase (CK) was elevated in both C57 and D2 *mdx* mice, leading to a statistically significant main effect of *mdx* genotype (Figure S1A). When examining gastrocnemius muscle and body mass, we found a significant main effect of background strain with D2 mice generally being smaller than C57 mice (Figures S1B and S1C). Furthermore, a significant interaction between background strain and *mdx* genotype showed that body mass was significantly lower in D2 *mdx* mice vs D2 WT mice (Figure S1C). In contrast, there were no differences in body mass with pairwise comparisons between C57 *mdx* and C57 WT mice. Figure 1D shows the gastrocnemius:body mass ratio, where a significant main effect of background strain indicated that this ratio was lower in mice on the D2 background compared with those on a C57 background (Figure S1D). Finally, holding impulse (hangwire time normalized to body mass) was significantly lower across both C57 *mdx* and D2 *mdx* mice compared with their respective WT groups as denoted by a significant main effect of *mdx* genotype (Figure S1E).

### Metabolic phenotyping of the C57 mdx and D2 mdx mice

Mice were also housed in the Promethion metabolic cage system for 48-h periods primarily to examine cage activity as another marker of whole-body muscle performance. Our data show that D2 *mdx* mice were less ambulant in their cages compared with D2 WT mice with the main effect of *mdx* genotype measured across the light, dark, and total daily phases (Figure S2A). However, we did not observe any such differences in cage activity with similar pairwise comparisons between C57 *mdx* mice and C57 WT mice (Figure S2B). Therefore, these findings are consistent with the earlier-onset muscle weakness and wasting previously found in D2 *mdx* mice (Coley et al., 2016; Hammers et al., 2020; van Putten et al., 2019).

Along with cage activity, we also looked for potential differences in daily energy expenditure. Our results show that D2 *mdx* mice, but not C57 *mdx* mice, had significantly elevated energy expenditure when compared to their respective WT groups (Figures S2C and S2D). This increase in daily energy expenditure in D2 *mdx* mice was met with a significant reduction in daily food intake compared with D2 WT mice (Figure S2E); however, no differences in food intake were found between C57 *mdx* and C57 WT mice (Figure S2F). There were no differences in water intake across any of the experimental groups (Figures S2G and S2H).

The Promethion metabolic cage system allows for certain behavioral analyses such as measuring the time the mice spent in or touching its “home”—a habitat enclosure on a slightly elevated platform. Our results show that D2 *mdx* mice spent less time in their home compared with D2 WT mice; however, this did not reach statistical significance (Figure S2I). There were also no differences detected between C57 *mdx* and C57 WT mice (Figure S2J). When examining the time mice were interacting or touching their home, we found that D2 *mdx* mice spent significantly less time performing this action compared with D2 WT mice (Figure S2K); however, no such difference was observed between C57 *mdx* and C57 WT mice (Figure S2L). As the home is on an elevated platform, we believe that this serves as an additional indication of muscle weakness, where D2 *mdx* mice were not strong enough to enter the elevated enclosure.

### Ca^2+^ uptake and sarco(endo)plasmic reticulum Ca^2+^-ATPase activity are impaired in D2 mdx mice but not in C57 mdx mice

After verifying the early onset of muscle weakness in D2 *mdx* mice, we then examined SERCA function in the gastrocnemius muscles across all experimental groups (Figure 1). Ca^2+^ uptake experiments revealed obvious impairments in both C57 and D2 *mdx* mice (Figure 1A). Traditionally, rates of Ca^2+^ uptake are examined using tangent analysis at a free Ca^2+^ concentration ([Ca^2+^]_free_) of 1000-2000 nM (Fajardo et al., 2015), however, for both C57 *mdx* and D2 *mdx* mice we could not utilize such approach as the Ca^2+^ uptake curves failed to cross 2000 and 1000 nM. This in and of itself is indicative of impaired SERCA function. Additionally, we examined the area-under-the-curve (AUC) and total Ca^2+^ taken into the SR during the 400s protocol (Figures 1B and 1C). For both parameters, we found a significant main effect of *mdx* genotype with elevated AUC and lowered total Ca^2+^ uptake, suggesting that C57 and D2 *mdx* mice have impaired SERCA function compared with their respective WT controls. However, we also found a significant interaction between background strain and *mdx* genotype that suggests that the elevation in AUC and reduction in total Ca^2+^ uptake is far more prominent in D2 *mdx* muscles (Figures 1B and 1C).

We then performed Ca^2+^-dependent SERCA activity assays in the gastrocnemius muscle (Figures 1D-1G). A significant main effect of the *mdx* genotype was detected for maximal SERCA activity, suggesting that both C57 and D2 *mdx* mice have lowered maximal rates of SERCA activity compared with their respective WT controls (Figure 1E). Further analysis revealed that the percent reduction in maximal SERCA activity was greater in D2 *mdx* (−20%) vs C57 *mdx* (−5%) mice (Figure 1F), which like our results with the Ca^2+^ uptake assay, is indicative of a more prominent impairment to SERCA function in D2 *mdx* mice vs C57 *mdx* mice. There were no differences in SERCA’s apparent affinity for Ca^2+^ which is measured by the *p*Ca_50_ or the [Ca^2+^]_free_ required to elicit ½ Vmax (Figure 1G).

To determine a potential pathological consequence of Ca^2+^ dysregulation, calpain activity was measured in the gastrocnemius. We found that calpain activity was significantly higher in the C57 and D2 *mdx* muscles compared with C57 and D2 WT as denoted by a significant main effect of *mdx* genotype (Figure 1H). In addition, a significant main effect of background strain indicated that D2 mice had greater calpain activity compared to C57 mice (Figure 1H). Together, this suggests that D2 *mdx* mice should have greater levels of calpain activity compared with C57 *mdx* mice; and while there was no significant interaction between background strain and *mdx* genotype, planned comparisons between D2 *mdx* and C57 *mdx* showed that calpain activity was significantly higher in D2 *mdx* mice (p = 0.0006, Student’s t-test).

### Elevated reactive oxygen and nitrogen species may contribute to impaired sarco(endo)plasmic reticulum Ca^2+^-ATPase function

**Figure 2:**
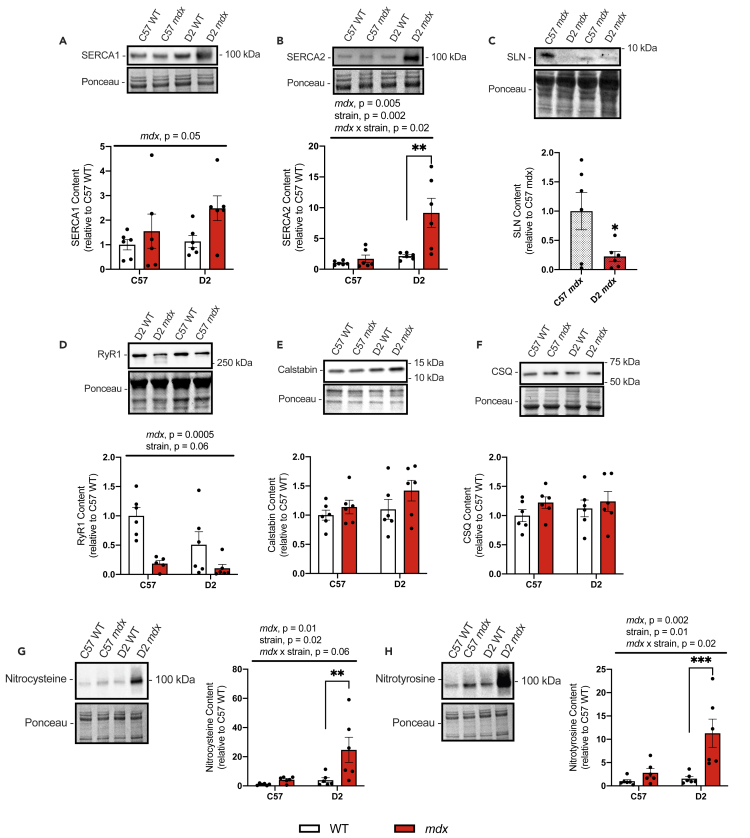
Western blot analyses of SR Ca^2+^ handling proteins, total protein nitrosylation, and tyrosine nitration in gastrocnemius muscles from C57- and D2 WT and *mdx* mice (A and B) SERCA1 and SERCA2 content. (C) SLN content in C57 and D2 *mdx* gastrocnemius muscles. (D–F) RyR1, CSQ, and calstabin content in gastrocnemius muscles from D2 and C57 -WT and -*mdx*. (G) Protein nitrosylation measured via western blotting for nitrocysteine-adducted protein. (H) Protein tyrosine nitration measured via western blotting for nitrotyrosine-adducted protein. For (A–C) and (D–H), two-way ANOVAs were performed with a Sidak’s multiple comparisons test, ∗∗p < 0.01, ∗∗∗p < 0.001, ∗∗∗∗p < 0.0001 n = 6 per group. Main effects and interactions expressed in bars over the graph. For C, a Student’s t test was used, ∗p < 0.05, n = 6 per group. All protein contents are expressed as arbitrary units and relative to C57 WT. Values are represented as mean ± SEM.

To examine the potential mechanisms explaining the drastic impairments in SERCA function found in D2 *mdx* gastrocnemius muscles, we performed western blotting for various SR Ca^2+^ handling proteins (Figure 2). First, we looked for potential differences in SERCA1 content. In this respect, we found a significant main effect of genotype, where SERCA1 was upregulated in *mdx* mice compared with WT mice (Figure 2A). Though we did not detect a statistically significant interaction between *mdx* genotype and background strain, it is worth noting that with a Student’s t-test, SERCA1 content was only significantly different for pairwise comparisons made between D2 *mdx* and D2 WT mice (p = 0.03) but not C57 *mdx* mice and C57 WT mice (p = 0.46). Similar results were found for SERCA2, where the main effect of *mdx* genotype suggested that *mdx* mice had more SERCA2 compared with WT mice; however, in this case, a significant interaction between *mdx* genotype and background strain indicated that the increase in SERCA2 was only statistically evident in D2 *mdx* mice and not C57 *mdx* mice (Figure 2B).

Next, we probed for sarcolipin (SLN)—a known inhibitor of SERCA that has been shown to be upregulated in muscles from C57 *mdx* mice (Fajardo et al., 2018; Schneider et al., 2013). We found that SLN was ectopically expressed in both the D2 *mdx* and C57 *mdx* gastrocnemius muscle as SLN could not be detected via western blot in the gastrocnemius muscles obtained from D2 WT or C57 WT mice (Figure S3). When comparing the levels of SLN protein between *mdx* models, we found that SLN content was significantly lower in D2 *mdx* gastrocnemius muscles compared with those from C57 *mdx* mice (Figure 2C).

RyR1 is the RyR isoform predominantly found in skeletal muscle and we found that content was lower in muscles obtained from C57 and D2 *mdx* mice compared with those obtained from C57 and D2 WT mice (Figure 2D). This was denoted with a significant main effect of the *mdx* genotype. There were no differences detected across any experimental groups when measuring the protein contents of calstabin, the channel stabilizer of RyR1, or calsequesterin (CSQ), the Ca^2+^ binding/buffering protein found in the SR (Figures 2E and 2F).

Finally, we measured the levels of total protein nitrosylation and nitration in the gastrocnemius via western blotting. As these blots were conducted under standard reducing conditions, they provide a measure of irreversible RONS modification. Our results show that the *mdx* mice (both C57 and D2) have elevated RONS in their gastrocnemius muscles compared with those from WT mice as we detected a significant main effect of *mdx* genotype for both protein nitrosylation and nitration (Figures 2G and 2H). Furthermore, we also found a significant main effect of background strain, where D2 mice had higher levels of protein nitrosylation and nitration compared with C57 mice (Figures 2G and 2H). Although the interaction between *mdx* genotype and background strain was only significant for protein nitrotyrosine, multiple comparisons across both protein nitrocysteine and nitrotyrosine datasets indicate that the increase in these markers of RONS was only statistically significant for D2 *mdx* mice vs D2 WT mice and not statistically significant in similar pairwise comparisons made between C57 *mdx* mice vs C57 WT mice (Figures 2G and 2H). Overall, this indicates that RONS is specifically elevated in young D2 *mdx* mice but not age-matched C57 *mdx* mice. This corresponds well with the differences in disease severity and could potentially contribute to the more drastic impairments in SERCA function observed in D2 *mdx* mice.

### Sarco(endo)plasmic reticulum Ca^2+^-ATPase function is also impaired in the D2 mdx diaphragm and left ventricle

**Figure 3:**
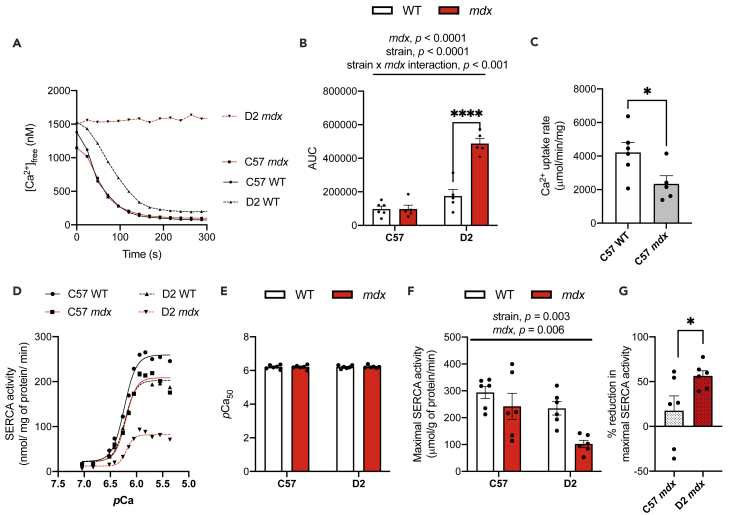
Impairments in Ca^2+^ uptake and maximal SERCA activity in diaphragm muscles from *mdx* mice are more prominent on the D2 background strain (A) Ca^2+^ uptake curves from diaphragm homogenates. (B) Area under the curve (AUC) of Ca^2+^ uptake curves. (C) Ca^2+^ uptake rate in diaphragm homogenates from C57 WT and *mdx* mice measured via tangent analysis at 1000 nM free [Ca^2+^]. (D) Ca^2+^-dependent SERCA activity curves in D2 and C57 -WT and -*mdx* diaphragm muscles. (E) *p*Ca_50_ of SERCA activity. (F) Maximal SERCA activity expressed as μmol/g of protein/min. (G) The % reduction in maximal SERCA activity in C57- and D2 *mdx* mice. For (B, E, and F), two-way ANOVAs were performed with a Sidak’s multiple comparisons test, ∗∗∗∗p < 0.0001 n = 5-6 per group. Main effects and interactions expressed in bars over the graph. Values are represented as mean ± SEM. For C and G, a Student’s *t* test was used, ∗p < 0.05.

Given the cardiorespiratory implications of DMD, we next assessed SERCA function in the diaphragm and cardiac (left ventricle) muscles from all experimental groups. Similar to our results with the gastrocnemius, we found that diaphragm muscles from D2 *mdx* mice had severe decrements in Ca^2+^ uptake with an obvious inability to lower [Ca^2+^]_free_ during the assay (Figure 3A). Additionally, we found a significant interaction between *mdx* genotype and background strain with our AUC analysis, indicating that D2 *mdx* mice had significantly elevated AUC compared with D2 WT mice (Figure 3B). Though AUC was not different between C57 *mdx* and C57 WT mice, we were able to measure Ca^2+^ uptake rates at a [Ca^2+^]_free_ of 1000 nM where we found a significantly slower rate of Ca^2+^ uptake in the diaphragm from C57 *mdx* mice compared with C57 WT mice (Figure 3C). This suggests that while Ca^2+^ uptake is impaired in the C57 *mdx* diaphragm, the extent of impairment is not as severe as that found in the D2 *mdx* diaphragm. Next, we measured SERCA activity (Figures 3D–3G). Similar to our findings in the gastrocnemius muscles, there were no changes in *p*Ca_50;_ however, a significant main effect of *mdx* genotype was detected for maximal SERCA activity suggests that C57 and D2 *mdx* mice have lowered maximal SERCA activity in their diaphragm (Figure 3F). Notably, the level of impairment was more prominent in the D2 *mdx* diaphragm (−56%) compared with the C57 *mdx* diaphragm (−17%; Figure 3G).

**Figure 4:**
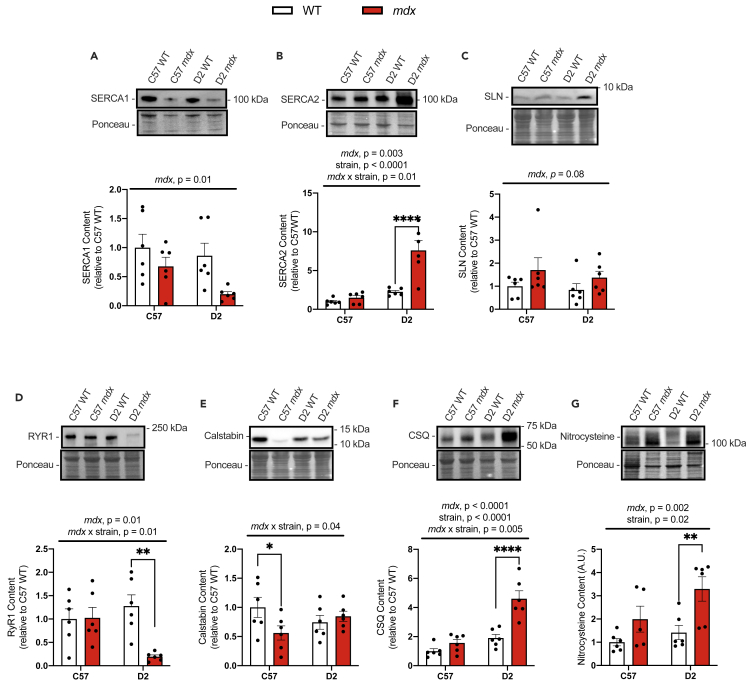
Western blot analyses of SR Ca^2+^ handling proteins and total protein nitrosylation in diaphragm muscles from C57- and D2 WT and *mdx* mice (A and B) SERCA1 and SERCA2 content. (C) SLN content in C57 and D2 WT and *mdx* diaphragm muscles. (D–F) RyR1, CSQ, and calstabin content in diaphragm muscles from D2 and C57 -WT and -*mdx*. (G) Protein nitrosylation measured via western blotting for nitrocysteine-adducted protein. Two-way ANOVAs were performed with a Sidak’s multiple comparisons test, ∗∗p < 0.01, ∗∗∗∗p < 0.0001 n = 6 per group. Main effects and interactions expressed in bars over the graph. All protein contents are expressed as arbitrary units and relative to C57 WT. Values are represented as mean ± SEM.

Western blot analysis of various SR Ca^2+^ handling proteins within the diaphragm revealed a significant reduction in SERCA1 content in both C57 and D2 *mdx* mice compared with their respective WT groups as denoted by a significant main effect of *mdx* genotype (Figure 4A). In contrast, there was a significant increase in SERCA2; however, this only occurred in D2 *mdx* mice vs D2 WT mice (Figure 4B). Furthermore, while SLN content appeared to be elevated in the diaphragm from both C57 and D2 *mdx* mice compared with their respective WT groups, the main effect of the *mdx* genotype was not statistically significant (Figure 4C). An interaction between *mdx* genotype and background strain was detected for RyR1, where its content was significantly reduced in the *mdx* diaphragm compared with WT, but only for D2 mice (Figure 4D). Another interaction was detected for calstabin, which showed that calstabin protein levels were reduced in the C57 *mdx* diaphragm compared with C57 WT mice; but no differences were found between D2 WT and D2 *mdx* mice (Figure 4E). Figure 4F illustrates yet another statistical interaction between *mdx* genotype and strain, in this case for CSQ, which suggests that CSQ protein content was only elevated in D2 *mdx* diaphragm muscles compared with D2 WT muscles. Finally, we found significant main effects of *mdx* genotype and background strain for total nitrosylation in the diaphragm (Figure 4G). Though there was no interaction between these two main effects of *mdx* genotype and background strain, planned comparisons show that only D2 *mdx* but not C57 *mdx* mice have significantly elevated protein nitrosylation compared with their respective WT groups.

**Figure 5:**
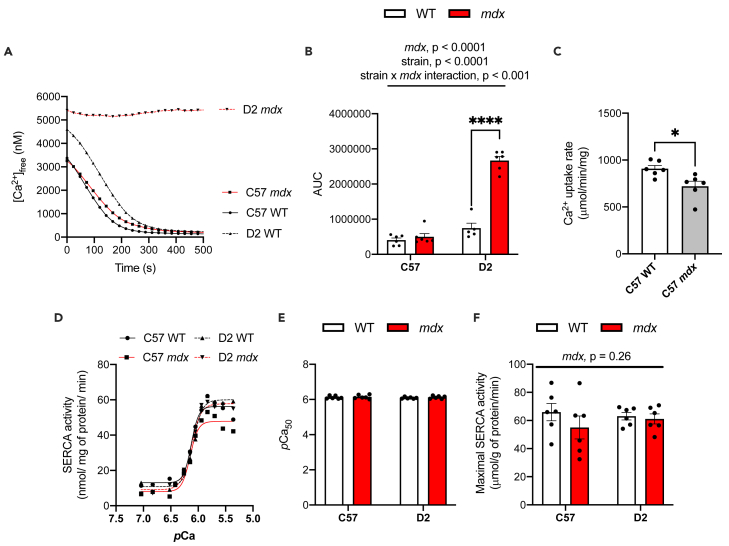
Impairments in Ca^2+^ uptake in left ventricles from *mdx* mice are more prominent on the D2 background strain (A) Ca^2+^ uptake curves from left ventricle homogenates. (B) Area under the curve (AUC) of Ca^2+^ uptake curves. (C) Ca^2+^ uptake rate in left ventricle homogenates from C57 WT and *mdx* mice measured via tangent analysis at 1000 nM free [Ca^2+^]. (D) Ca^2+^-dependent SERCA activity curves in left ventricle homogenates from D2 and C57 -WT and -*mdx* mice. (E) *p*Ca_50_ of SERCA activity. (F) Maximal SERCA activity expressed as μmol/g of protein/min. For (B, E, and F), two-way ANOVAs were performed with a Sidak’s multiple comparisons test, ∗∗∗∗p < 0.0001 n = 5-6 per group. Main effects and interactions expressed in bars over the graph. Values are represented as mean ± SEM. For C, a Student’s t test was used, ∗p < 0.05.

**Figure 6:**
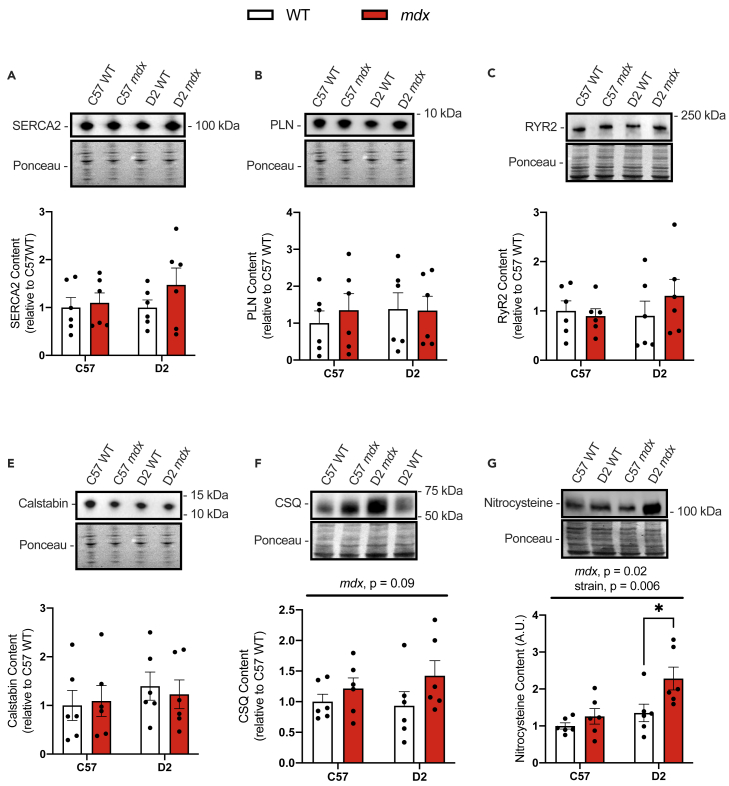
Western blot analyses of SR Ca^2+^ handling proteins and total protein nitrosylation in left ventricles from C57- and D2 WT and *mdx* mice (A) SERCA2 content in left ventricle homogenates. (B) PLN content in C57 and D2 WT and *mdx* diaphragm muscles. (C–F) RyR2, calstabin, and CSQ content in left ventricle homogenates from C57 and D2 -WT and -*mdx* mice. (G) Protein nitrosylation measured via western blotting for nitrocysteine-adducted protein. Two-way ANOVAs were performed with a Sidak’s multiple comparisons test, ∗p < 0.05, n = 6 per group. Main effects and interactions expressed in bars over the graph. All protein contents are expressed as arbitrary units and relative to C57 WT. Values are represented as mean ± SEM.

In the left ventricle, we again found obvious impairments in Ca^2+^ uptake ability in the D2 *mdx* mice leading to a significantly higher AUC compared with D2 WT mice (Figures 5A and 5B). Similar to the diaphragm, tangent analysis at 1000 nM [Ca^2+^]_free_ revealed a significant reduction in Ca^2+^ uptake rates in the C57 *mdx* heart compared with hearts obtained from C57 WT mice (Figure 5C). This again suggests that while Ca^2+^ uptake is impaired in both C57 and D2 *mdx* hearts, the level of impairment is more severe in the latter. We did not find any differences in maximal SERCA activity or *p*Ca_50_ across the experimental groups (Figures 5D–5F), nor in our western blot analysis of SERCA2, phospholamban (PLN; a well-known SERCA inhibitor found in the heart), RyR2 (cardiac isoform of RyR), calstabin, or CSQ (Figures 6A–6F). However, similar to our findings with the gastrocnemius and diaphragm muscles, we found a significant main effect of *mdx* genotype and background strain for total protein nitrosylation (Figure 6G). This suggests that the *mdx* genotype and the D2 background led to greater levels of protein nitrosylation in the left ventricle. Furthermore, planned comparisons revealed that only the D2 *mdx* left ventricle had elevated protein nitrosylation compared with D2 WT, whereas there were no significant differences detected with similar pairwise comparisons made between C57 WT and C57 *mdx* mice (Figure 6G).

## Discussion

To our knowledge, this study is the first to compare SERCA function in skeletal and cardiac muscles from both the D2 *mdx* and C57 *mdx* mouse models. The D2 *mdx* mouse is known as a more severe model of DMD with early onset muscle weakness compared to the C57 *mdx* mouse. Our present findings with hangwire impulse and cage ambulation are in support of this and show that D2 *mdx* mice had early onset muscle weakness occurring at 8-10 weeks of age. The D2 *mdx* mice also interacted less with the elevated mouse home enclosure compared to C57 *mdx* mice, suggesting that these mice were less able to jump onto this raised platform found within the Promethion metabolic cages. With respect to SERCA, our findings show that SERCA function, particularly Ca^2+^ uptake and maximal SERCA activity, is impaired across most muscles from both C57 and D2 *mdx* mice; however, this effect is more prominent in the latter. This is not only consistent with our original hypothesis but is also consistent with a previous study showing that SERCA dysfunction proceeds on a continuum of disease severity, where more severe dystrophic models such as the dko mouse display larger impairments in SERCA function (Schneider et al., 2013).

Across all muscles analyzed (gastrocnemius, diaphragm, and left ventricle), we found that Ca^2+^ uptake was impaired in both C57 and D2 *mdx* mice. The impairments in Ca^2+^ uptake observed in the C57 *mdx* mice were not entirely surprising given that this has been found in other previous studies (Schneider et al., 2013; Wasala et al., 2020). However, in D2 *mdx* mice, the level of impairment in Ca^2+^ uptake ability was so drastic that there was very little uptake occurring in the gastrocnemius, diaphragm, and left ventricle, ultimately manifesting as a larger AUC. Similar to our results with Ca^2+^ uptake, the reductions in maximal SERCA activity were more severe in muscles from D2 *mdx* mice compared with C57 *mdx* mice—at least for the gastrocnemius and diaphragm muscles. We did not find any differences in maximal SERCA activity in either the C57 or D2 *mdx* left ventricle than their respective WT groups and this is perhaps in line with previous findings, indicating that cardiac impairments manifest at a much later age for both C57 and D2 *mdx* mice (Coley et al., 2016; van Putten et al., 2019). This may be at odds with our Ca^2+^ uptake data, especially in the D2 *mdx* left ventricle, where there were clear and obvious impairments in Ca^2+^ uptake ability. It is possible that of the two assays, Ca^2+^ uptake is more affected than SERCA activity in D2 *mdx* mice. The elevated oxidative stress found presently in the D2 *mdx* cardiac (and skeletal) muscle could be affecting other SR-related proteins (i.e., RyR) and lipids that could contribute to enhanced SR Ca^2+^ leak thereby disrupting the net Ca^2+^ uptake rate. This could provide an explanation for as to why Ca^2+^ uptake was greatly impaired in the D2 *mdx* left ventricle despite there not being any difference in maximal SERCA activity. In fact, an increase in Ca^2+^ leak could lead to an increase in SERCA-mediated ATP hydrolysis. This can be seen in cases of malignant hyperthermia where genetically susceptible individuals will experience a hypermetabolic state while under anesthesia owing to excessive Ca^2+^ leak from the RyR. Indeed, we also found that D2 *mdx* mice had the highest levels of daily energy expenditure, and we speculate that this may be owing to some inefficiencies of the SERCA pump, where the amount of Ca^2+^ was brought into the SR for every ATP hydrolyzed is effectively lowered. However, future studies aimed at specifically quantifying the SERCA coupling ratio in these muscles are required. It should also be noted that a previous study found that daily energy expenditure was elevated in 5-month-old C57 *mdx* mice compared with age-matched C57 WT mice (Strakova et al., 2018). Though we did not make such an observation in this present study, we believe that this discrepancy could possibly be explained by differences in age. That is, the C57 *mdx* mice used in this study were younger compared to the mice used in the study by Strakova and colleagues. Perhaps in our hands, we would observe a “hypermetabolic” state in older C57 *mdx* mice.

The alterations to SERCA pump function, particularly in D2 *mdx* mice, do not seem to be explained by differences in SERCA protein levels. Both SERCA1 and SERCA2 were upregulated in the gastrocnemius, which we believe is an adaptive response aimed at improving SR Ca^2+^ handling as SERCA density is an important determinant of SR Ca^2+^ filling, at least in healthy conditions (Tupling, 2004). However, any compensatory increase in SERCA content/density was unsuccessful in rescuing SERCA function and our analyses do not discriminate between functional and non-functional SERCA protein. Conversely, and in the diaphragm, SERCA1 protein was found to be reduced across both *mdx* models, and SERCA2 was upregulated only in the D2 *mdx* mouse (vs D2 WT). Although the exact reasons for the downregulation of SERCA1 in the *mdx* diaphragm are unclear, it is possible that this may be reflective of a fast-to-slow fiber type shift that has occurred across both *mdx* models. Calcineurin is a well-known promoter of the oxidative myogenic program and has been shown to be more active in *mdx* mice, especially in their diaphragm (Stupka et al., 2006). Finally, in the left ventricle, we did not observe any changes in SERCA2 content.

SLN is a small peptide that binds to and inhibits the SERCA pump; and a previous study in C57 *mdx* and dko mice has shown that SLN protein is elevated in accordance with disease severity with greater levels of SLN found in muscles from dko mice compared with *mdx* mice (Schneider et al., 2013). Moreover, SLN is a well-known uncoupler of the SERCA pump that increases the energetic requirements of Ca^2+^ transport (Bombardier et al., 2013; Smith et al., 2002), and therefore, increased SLN expression in muscle could have partly explained the hypermetabolic phenotype observed in D2 *mdx* mice. However, while SLN protein was elevated in the *mdx* gastrocnemius and diaphragm muscles, this did not occur to a greater extent in the D2 *mdx* mouse vs C57 *mdx* mouse. In fact, in the gastrocnemius, the level of SLN upregulation was greater in C57 *mdx* mice vs D2 *mdx* mice. SLN has also been implicated in *mdx* pathology with the genetic ablation of SLN leading to improvements in skeletal and cardiac muscle health/function (Balakrishnan et al., 2022; Mareedu et al., 2021; Tanihata et al., 2018; Voit et al., 2017). However, we were not able to reproduce these findings and found that the genetic deletion of *Sln* worsened the disease by limiting calcineurin activation (Fajardo et al., 2018). Calcineurin is not only important for promoting the oxidative myogenic program but also for resisting dystrophic pathology (Chakkalakal et al., 2004; Stupka et al., 2008). Thus, there is a clear discrepancy in the literature regarding the role of SLN in mediating dystrophic pathology. Although we do not know the exact cause for these discrepant findings, we believe that differences in SERCA function in the face of *Sln* deletion may be at play (Chambers et al., 2022). In any event, given the novelty of the D2 *mdx* mouse and its apparent applicability to the human condition, future studies examining the effects of *Sln* deletion on the D2 *mdx* mouse, perhaps with a multicentered approach (i.e., across various institutions), could be of value.

We next investigated the protein levels of other SR-related proteins that could indirectly affect SERCA function in muscle, namely RyR (RyR1 in gastrocnemius and diaphragm muscles; and RyR2 in cardiac muscles), calstabin, and CSQ. Changes in RyR Ca^2+^ release and/or leak could indirectly impair SERCA by countering the effects of SERCA-mediated Ca^2+^ transport. In the D2 *mdx* gastrocnemius and diaphragm muscles, and in the C57 *mdx* gastrocnemius muscle, we found lowered RyR1 content compared with their respective WT groups. These results are inconsistent with a previous study that found no differences in RyR1 content in *mdx* mice vs WT mice across a variety of ages (Bellinger et al., 2009). Furthermore, while Bellinger et al. (2009) found no differences in the calstabin content between C57 WT and C57 *mdx* mice, we found that calstabin was lower in the C57 *mdx* diaphragm compared with C57 WT diaphragm. Calstabin stabilizes RyR in a closed state and its reduction could indirectly contribute to the impairments in Ca^2+^ uptake observed in the C57 *mdx* diaphragm. Although we do not have an explanation for these discrepant findings between studies, we note that the reduction in RyR1 observed in the present study could signify a reduction in full-length RyR1 and an increase in its fragmentation. Reductions in full-length RyR1 in human skeletal muscle have been observed in a recent study in response to sprint interval training and were taken as a possible indication of increased RyR1 fragmentation (Tripp et al., 2022) based on results from other previous studies (Place et al., 2015; Schlittler et al., 2019). This is important as reductions in full-length RyR1 and increased RyR1 fragmentation has been linked to SR Ca^2+^ leak (Place et al., 2015). Although it is tempting to speculate that this may also be occurring in *mdx* skeletal muscles, future studies devoted to RyR1 and RyR2 Ca^2+^ leak in the D2 *mdx* skeletal and cardiac muscle are required. There were no changes in CSQ content in the gastrocnemius or left ventricle; however, there was a significant upregulation in CSQ in the D2 *mdx* diaphragm compared with WT. CSQ regulates the free Ca^2+^ levels in the SR and can indirectly promote SERCA function by reducing back-inhibition. However, the upregulation in CSQ found in the diaphragm of D2 *mdx* mice was associated with severe impairments in Ca^2+^ uptake and SERCA activity and could therefore signify a failed compensatory adaptation.

To further examine the cellular mechanisms behind the more severe impairments in SERCA function found in muscles from D2 *mdx* mice, we assessed levels of protein nitrosylation and/or nitration as markers of elevated oxidative and nitrosative stress. Across the gastrocnemius, diaphragm, and left ventricle, we found significant elevations in protein nitrosylation and/or nitration in D2 *mdx* compared with D2 WT mice, whereas these differences were not statistically significant in C57 *mdx* mice vs C57 WT mice. These results suggest that young D2 *mdx* mice, but not age-matched C57 *mdx* mice, present with signs of early-onset oxidative/nitrosative stress. This is important as elevated RONS can impair SERCA function (Viner et al., 1996, 1997, 1999a, 1999b), thereby providing a potential mechanism that could explain the severe impairments in SERCA function found in D2 *mdx* mice. It should also be noted that SERCA dysfunction and the ensuing impairments in cytosolic Ca^2+^ regulation could also negatively affect mitochondrial function leading to increased production of RONS and the manifestation of a vicious cycle where SERCA impairments can lead to RONS production, and RONS can impair SERCA function.

Another pathological consequence of impaired SERCA function and dysregulated cytosolic Ca^2+^ levels is the over-activation of calpain-mediated proteolysis. To our knowledge, ours is the first study to demonstrate that rates of calpain activity are higher in muscles from mice on a D2 genetic background compared with mice on a C57 genetic background. Furthermore, planned comparisons between D2 *mdx* and C57 *mdx* mice specifically revealed a significant 2.2-fold increase in calpain activity in the former vs the latter (p = 0.0006 via Student’s *t* test). These findings provide additional mechanistic insight that may partly explain the worsened pathology found in the D2 *mdx* mouse.

In conclusion, our study clearly shows drastic impairments in SERCA function in skeletal and cardiac muscles from D2 *mdx* mice. These results suggest that the early onset muscle weakness and damage observed in young D2 *mdx* mice may be partly owing to an inability to bring Ca^2+^ into the SR. Future studies should investigate whether improving SERCA function, potentially through cytotoxic protection against RONS may mitigate dystrophic pathology and improve physiological outcomes in the D2 *mdx* mouse.

### Limitations to the study

We acknowledge that our work is largely descriptive, and future studies aimed at improving SERCA function will determine its causal role, if any, in D2 *mdx* pathology. The mechanisms leading to these impairments in SERCA function are still unknown, and though we associate them with elevated oxidative/nitrosative stress, SERCA-specific modifications should be assessed in the future. RyR Ca^2+^ leak is another major player in the Ca^2+^ disturbances found in C57 *mdx* muscle (Bellinger et al., 2009; Capogrosso et al., 2018), and therefore, our study is limited in that we did not examine RyR function or SR Ca^2+^ leak. Finally, our study does not discern whether the elevated daily energy expenditure in D2 *mdx* mice is caused solely by increased muscle-based energy consumption. Other thermogenic organs such as brown and beige fat could be investigated in the future.

## STAR★Methods

### Key resources table

**Table:** 

REAGENT or RESOURCE	SOURCE	IDENTIFIER
**Experimental models: Organisms/strains**		

Mouse: C57BL/10ScNJ	The Jackson Laboratories	Strain #:**003752**
Mouse: C57BL/10ScSn-*Dmd*^*mdx*^/J	The Jackson Laboratories	Strain #:**001801**
Mouse: D2.B10-*Dmd*^*mdx*^/J	The Jackson Laboratories	Strain #:**0013141**
Mouse: DBA/2J	The Jackson Laboratories	Strain #:**000671**

**Software and algorithms**		

Molecular Devices SoftMax Pro	Molecular Devices	%%%https://www.moleculardevices.com/products/microplate-readers/acquisition-and-analysis-software/softmax-pro-software?cmp=7010g000000nNCH&utm_source=AdWords&utm_medium=cpc&utm_campaign=MPR-Brand_CANADA&utm_adgroup={adgroup}&utm_location=9000776&utm_keyword=molecular%20devices%20softmax%20pro&utm_device=c&utm_devicemodel=&utm_placement=&utm_adpostion=&utm_target=&utm_network=g&utm_creative=413034758091&gclid=CjwKCAjw0dKXBhBPEiwA2bmObfQYfxhI9dqx2kDoEaENs2bFYuBzCML-NluCxhjnZ3efLgFFif0rvhoCsnYQAvD_BwE
ImageLab	BioRad	https://www.bio-rad.com/fr-ca/product/image-lab-software?ID=KRE6P5E8Z
Graphpad Prism 8	Graphpad Prism	https://www.graphpad.com/scientific-software/prism/

### Resource availability

#### Lead contact

Further information and requests for resources and reagents should be directed to and will be fulfilled by the lead contact, Val A. Fajardo (vfajardo@brocku.ca).

#### Materials availability

This study did not generate new unique reagents.

#### Data and code availability

All data are included in the published article or are available from the lead contact upon reasonable request. This paper does not report original code.

### Experimental models and subject details

#### Mice and Design

Male C57 *mdx* (n = 12), C57 WT (n = 12), D2 *mdx* (n = 12), and D2 WT (n = 12) mice were purchased from Jackson Laboratories at 7–8 weeks of age. They were acclimated and housed in Brock University’s Animal Facility, in an environmentally controlled room with a standard 12:12 h light-dark cycle and allowed access to food and water *ad libitum*. After the mice reached 9–10 weeks of age, they were euthanized via cervical dislocation under general anesthetic (vaporized isoflurane) and their tissues were collected.

#### Ethics statement

All animal procedures were approved by the Brock University Animal Care and Utilization Committee (file #17-06-03) and were carried out in accordance with the Canadian Council on Animal Care guidelines.

### Method details

#### Hang wire testing

At 8–9 weeks of age, mice were subjected to a hang wire test to determine limb strength and endurance. All mice were gently placed on the wire situated 12 inches high and were left suspended on the wire until they reached exhaustion and dropped from the wire to the base of the cage. The time they remained suspended was recorded for three trials, separated by a 60s recovery period. Impulse (s∗g) was calculated according to DMD_M.2.1.004 standard operating procedures by multiplying the average time suspended by body mass.

#### Metabolic caging

At 8–9 weeks of age, mice were housed in pairs in a Promethion Metabolic Cage System for 48 h. Two 12-h light and dark cycles were measured, and data was collected. Food and water intake were measured through mass changes with the MM-1 load cell, the Promethion mass measurement device. Cage ambulation was quantified through metres travelled which is collected through beam breaks with the BXYZ Beambreak Activity Monitor in the x y, and z planes. Respiratory exchange ratio (RER) was calculated as VCO_2_ (volume of carbon dioxide expired each minute) divided by VO_2_ (volume of oxygen inspired each minute) measured from the Promethion metabolic cages equipped with O_2_ and CO_2_ gas analyzers. To obtain mean energy expenditure expressed in kcal/h, the Weir equation was used (41).

#### Sample collection and homogenization

Gastrocnemius, diaphragm, and cardiac muscles (n = 12/group) were rapidly extracted from euthanized animals and flash frozen in liquid N2 and stored at −80°C. For Ca^2+^ uptake and SERCA activity analyses, muscles were homogenized in homogenizing buffer (5 mM HEPES, 250 mM sucrose, 0.2 mM PMSF, 0.2% NaN3; pH 7.5). An aliquot of the muscle homogenate was then supplemented with protease and phosphatase (phosSTOP; cOmplete Mini) inhibitors for western blot analyses. Blood was collected via cardiac puncture and spun at 5000 × g for 8 min (4°C) and serum was collected and stored at −80°C.

#### Serum creatine kinase activity

Serum creatine kinase activity was determined as previously described (42), with a M2 Molecular Device plate reader and a commercially available assay (Cat. #C7522, Pointe Scientific Inc., Canton, MI, USA) fitted onto a 96-well plate and calibrated with a standard curve of purified creatine kinase (Sigma, Oakville, ON, Canada, Cat. 10127566001).

#### Ca^2+^ uptake

Ca^2+^ uptake assays were fitted onto a 96-well plate and done on a M2 Molecular Device plate reader and the ratio-able Ca^2+^ fluorescent indicator Indo-1. In brief, 20 μL of diaphragm, 25 μL of gastrocnemius or 50 μL of left ventricle homogenate was added to 200 μL Ca^2+^ uptake buffer (20 mM HEPES, 200 mM KCL, 10 mM NaN_3_, 5 μM TPEN, 15 mM MgCl_2_, 5 mM Oxalate; pH 7.0), and 1 μL of Indo-1 (1 mM in 50 mM glycine, pH 7.0). Ca^2+^ uptake was initiated with 4 μL of ATP (250 mM, pH 7.0) with the plate read kinetically set with an excitation wavelength of 355 nM and emission wavelengths of 405 nm (Ca^2+^-bound Indo-1) and 485 nm (Ca^2+^-free Indo-1). Free Ca^2+^ concentration was calculated using the following formula and a Kd of 250 nM for Indo-1. The amount of Ca^2+^ uptake were measured through an area under the curve analysis (AUC). In addition, rates of Ca^2+^ uptake were analyzed and normalized to wet tissue weight in mg.

$$\left[Ca^{2\;+}\right]=K_{d}\;\left(\frac{R-\;R_{min}}{R_{max}-R}\right)\left(\frac{S_{f2}}{S_{b2}}\right)$$

Formula 1: Free Calcium Concentration (*[Ca*^*2+*^*]*_*f*_)

*K*_*d*_ is the dissociation constant of Indo-1 (250 nM). *R*_*min*_ refers to the ratio of bound:unbound Indo-1 after adding 15 mM EGTA. *R*_*max*_ refers to the ratio of bound:unbound Indo-1 after adding 5 M CaCl_2_. *S*_*f2*_ is the fluorescence emission at 485 nm (Ca^2+^-free Indo-1) in the EGTA portion and *S*_*b2*_ is the fluorescence emission at 485 nm in the 5 M CaCl_2_ portion.

#### SERCA ATPase activity

Ionophore (A23187; Sigma Aldrich) supported SERCA activity was measured in gastrocnemius homogenates using an enzyme-linked spectrophotometric assay as previously described (24, 30, 43). Briefly, muscle homogenates (15 μL), pyruvate kinase (PK) and lactate dehydrogenase (LDH) (18 U I^−1^ for both) were added to Ca^2+^ ATPase buffer (20 mM HEPES, 200 mM KCl, 10 mM NaN3, 1 mM EGTA, 15 mM MgCl2, 5 mM ATP, 10 mM phosphoenolpyruvate). To initiate SERCA activity, 4 μL of 1.9% (w/v) NADH was added and the plates were then read at 340 nm for 30 min. SERCA-dependent activity in gastrocnemius homogenates was calculated with a pathlength correction, extinction coefficient of NADH (6.22 mM), and the subtraction of ATPase activity in the presence of 1 μL SERCA-specific inhibitor, CPA (40 mM) from total ATPase activity across a range of Ca^2+^ concentrations (*p*Ca 5.02–7.04). A bicinchoninic acid (BCA) assay was done to normalize SERCA activity to grams of protein.

#### Western blotting

Western blotting was conducted to determine protein expression of SERCA1/2, SLN, PLN, RYR, CSQ, calstabin, and levels of protein nitrosylation and nitration. Laemmli buffer was added to muscle homogenates to solubilize proteins that were then electrophorectially separated at 240 V for 22 min on a 7–12% TGX gradient gel (BioRad). Proteins were transferred to PVDF or nitrocellulose membranes for 6 min using BioRad Trans Blot Turbo. Prior to blocking, SuperSignal western Blot Enhancer (46641) from Thermo Scientific was applied for the SLN blot. A 5% (w/v) milk and TBST solution was used to block the membranes for one hour. Primary antibody was then added and incubated overnight at 4°C (see Table S1 for primary antibody, secondary antibody, and protein load details). Subsequent to primary incubation, the membranes were washed three times with TBST and then incubated with anti-mouse or anti-rabbit secondary antibodies at room temperature for 1 h. The membranes were then washed again three times with TBST then chemiluminescent substrate Millipore Immobilon (WBKLS0500; Sigma-Aldrich) or SuperSignal West Femto Maximum Sensitivity Substrate (34095; Thermo Scientific) was added prior to imaging with a BioRad Chemidoc. Optical densities were analyzed with imageLab (BioRad) and normalized to total protein visualized with a ponceau stain (59803; Cell Signalling Technology).

#### Calpain activity assay

A commercialized calpain assay (QIA120; Millipore Sigma) was used in order to determine calpain activity in the gastrocnemius. Tissue was freshly homogenized in RIPA lysis buffer and 50 μL of sample was loaded in duplicate in either 100 μL of activator buffer or 100 μL of inhibitor buffer in a 96-well plate as per the manufacturer’s instructions. Then, 50 μL of diluted substrate was added in the dark into each well and the plate was incubated for 15 min. The plate was then measured for 10 min as a kinetic plate at an excitation of 370 nm and emission of 450 nm to determine the rate of calpain activity normalized to total protein (via BCA assay).

### Quantification and statistical analysis

#### Statistical analysis

A two-way ANOVA was used to test the main effects of genotype (*mdx*) and strain (C57 or D2) and their potential interaction for most analyses. Post-hoc testing and planned comparisons (total protein nitrosylation in gastrocnemius, diaphragm, and left ventricle) was completed using Sidak multiple comparison testing. A Student’s t-test was also used to make specific comparisons between C57 and D2 *mdx* mice (i.e., calpain activity, percent reductions in maximal SERCA activity, and SLN content in the gastrocnemius muscle), C57 *mdx* and C57 WT mice (Ca^2+^ uptake rates for the diaphragm and left ventricle), as well as for some of the Promethion metabolic cage data (time spent in or touching the home). Statistical tests were conducted using GraphPad Prism 8 Software (San Diego, USA). All values are presented as means ± standard error. Statistical significance is set to p ≤ 0.05.
