# Supplementary material for: Sarco(endo)plasmic reticulum Ca2+-ATPase function is impaired in skeletal and cardiac muscles from young DBA/2J mdx mice
Source: iScience. 2022 Aug 18;25(9):104972. doi: 10.1016/j.isci.2022.104972 (PMC9459692; doi:10.1016/j.isci.2022.104972)
Supplement: Document S1. Figures S1–S3 and Table S1 [file mmc1.pdf]

iScience, Volume *25*

Supplemental information

Sarco(endo)plasmic reticulum Ca^{2+}-ATPase function is impaired in skeletal and cardiac muscles from young DBA/2J mdx mice

Riley E.G. Cleverdon, Jessica L. Braun, Mia S. Geromella, Kennedy C. Whitley, Daniel M. Marko, Sophie I. Hamstra, Brian D. Roy, Rebecca E.K. MacPherson, and Val A. Fajardo

SERCA function is impaired in skeletal and cardiac muscles from young DBA/2J mdx mice

Riley EG Cleverdon[1,2], Jessica L Braun[1,2], Mia S. Geromella[1,2], Kennedy C Whitley[1,2], Daniel M Marko[3], Sophie I Hamstra[1,2], Brian D Roy[1,2], Rebecca EK MacPherson[3,4], Val A Fajardo[1,2,4]*

SUPPLEMENTARY INFORMATION

Supplementary Table 1. Western blot details for across gastrocnemius, diaphragm, and left ventricle tissue. Related to Figures 2, 4 and 6.

**Target protein**	**Protein**	**Primary antibody supplier (catalog #)**	**Primary dilution**	**Secondary antibody supplier (catalog #)**	**Secondary dilution**
Gastrocnemius					
SERCA1	1.5 ug	Invitrogen MA3-911<br>RRID: AB_325494	1:2000	Cell Signalling 7076s	1:10000
SERCA2	1.5 ug	Invitrogen MA3-919<br>RRID: AB_325502	1:2000	Cell Signalling 7076s	1:10000
SLN	24 ug	EMD Millipore ABT13<br>RRID: AB_11203316	1:500	Cell Signalling 7074s	1:2000
RyR1	10.5 ug	Invitrogen MA3-925<br>RRID: AB_2254138	1:1000	Cell Signalling 7076s	1:2000
Calstabin	7.5 ug	Invitrogen PA1-026A<br>RRID: AB_2102731	1:1000	Cell Signalling 7074s	1:5000
CSQ	15 ug	Invitrogen MA3-913<br>RRID: AB_325496	1:2000	Cell Signalling 7076s	1:5000
Nitrocysteine	7.5 ug	Abcam ab94930	1:2000	Cell Signalling 7076s	1:10000
Nitrotyrosine	7.5 ug	Cayman chemical 189542	1:2000	Cell Signalling 7076s	1:10000
Diaphragm					
SERCA1	5 ug	Invitrogen MA3-911<br>RRID: AB_325494	1:5000	Cell Signalling 7076s	1: 20000
SERCA2	5 ug	Invitrogen MA3-919<br>RRID: AB_325502	1:5000	Cell Signalling 7076s	1:20000
SLN	25 ug	EMD Millipore ABT13<br>RRID: AB_11203316	1:200	Jackson ImmunoResearch 711-035-152	1:2000
RyR1	10 ug	Invitrogen MA3-925<br>RRID: AB_2254138	1:2000	Cell Signalling 7076s	1:2000
Calstabin	10 ug	Invitrogen PA1-026A<br>RRID: AB_2102731	1:2000	Cell Signalling 7074s	1:5000
CSQ	10 ug	Invitrogen MA3-913<br>RRID: AB_325496	1:2000	Cell Signalling 7076s	1:5000
Nitrocysteine	15 ug	Abcam ab94930	1:2000	Cell Signalling 7076s	1:2000
Left ventricle					
SERCA2	2.5 ug	Invitrogen MA3-919<br>RRID: AB_325502	1:5000	Cell Signalling 7076s	1:20000
PLN	2.5 ug	Invitrogen MA3-922<br>RRID: AB_2252716	1:2000	Jackson ImmunoResearch 711-035-152	1:2000
RyR2	15 ug	Invitrogen PA5-87416<br>RRID: AB_2804131	1:2000	Cell Signalling 7074s	1:2000
Calstabin	15 ug	Invitrogen PA1-026A<br>RRID: AB_2102731	1:2000	Cell Signalling 7074s	1:5000
CSQ	15 ug	Invitrogen MA3-913<br>RRID: AB_325496	1:2000	Cell Signalling 7076s	1:5000
Nitrocysteine	15 ug	Abcam ab94930	1:2000	Cell Signalling 7076s	1:2000

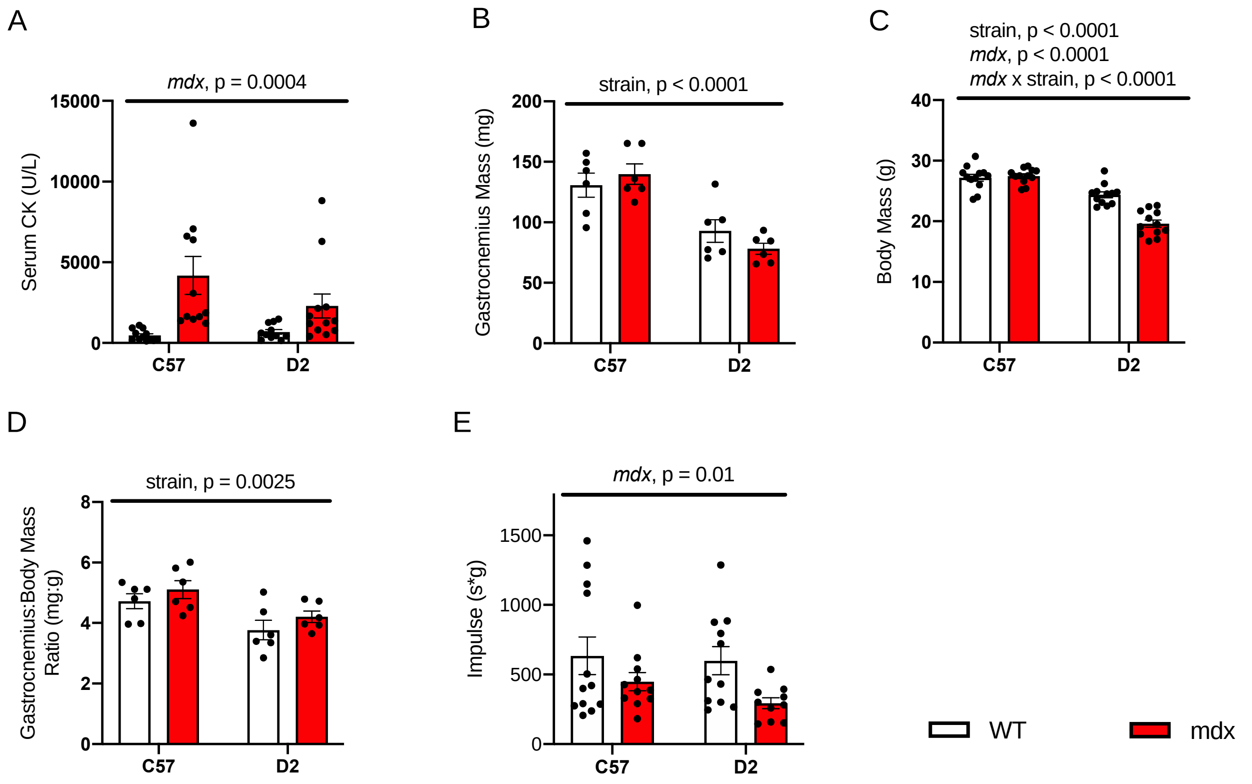

Supplementary Figure 1. Creatine kinase, muscle/body mass and impulse. Related to Figures 1 and 2. A) Serum creatine kinase activity expressed in units per litre. B) Gastrocnemius mass (mg), C) body mass (g), and D) gastrocnemius mass:body mass ratio (mg:g). E) Impulse expressed as hangwire time*body mass. A two-way ANOVA was used for all comparisons, n=12 per group (except for B and D, n = 6 per group). Values are represented as mean ± SEM. Main effects and interactions expressed in bars over the graph, ****$p < 0.0001$ using a Sidak's post-hoc test.

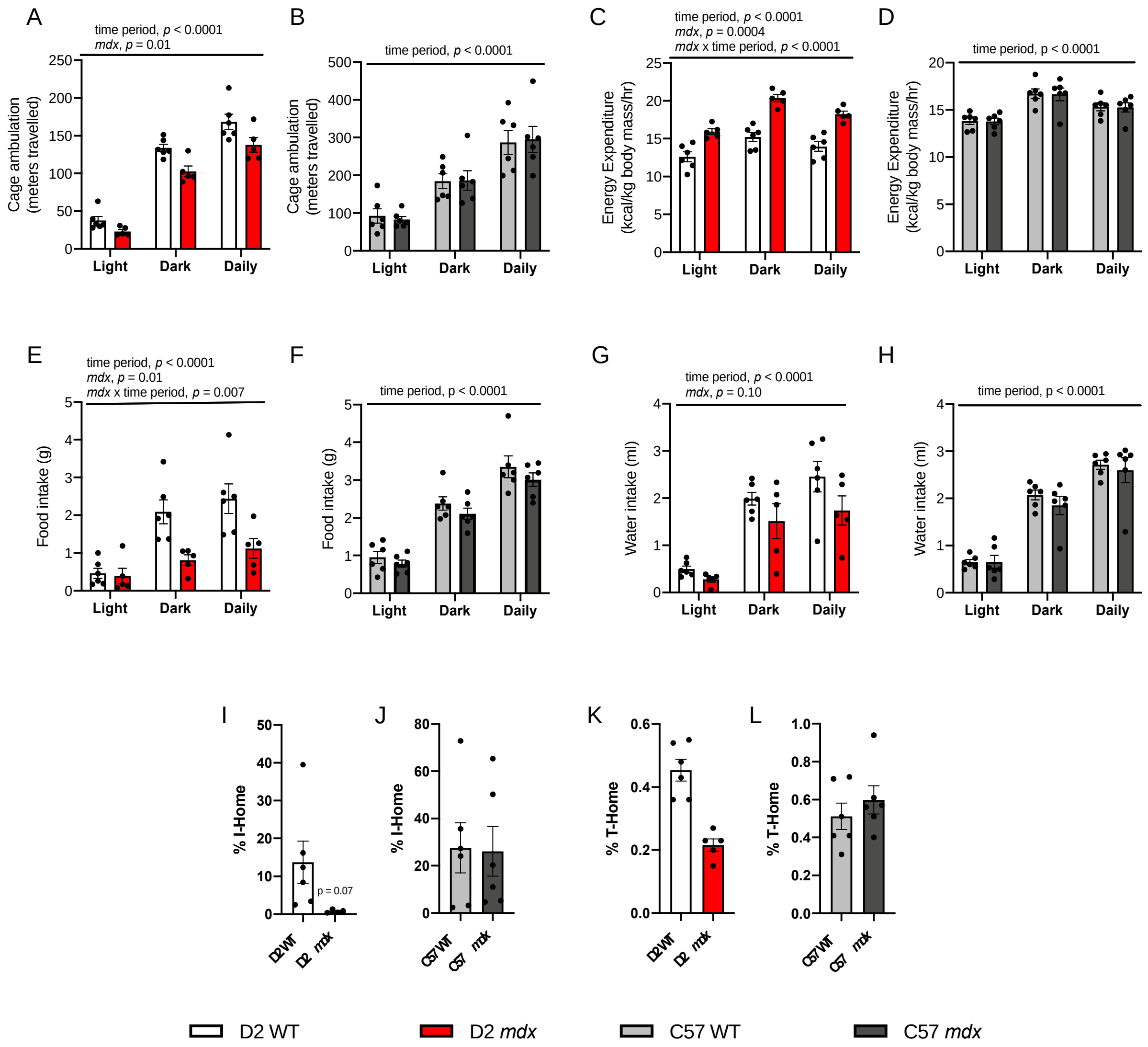

Supplementary Figure 2. Cage activity, energy expenditure and cage behaviour. Related to Figures 1-6. A) Daily cage ambulation in D2 mice and B) C57 mice expressed in metres travelled. C) Energy expenditure in D2 mice and D) C57 mice expressed in kcal/kg of body mass/hr. E) and G) Food (g) and water (mL) intake of D2 mice and F) and H) C57 mice. Percentage of time spent in (I-Home) and interacting with (T-Home) the mouse home for D2 mice I) and K) and C57 mice J) and L). All metabolic cage analyses were measured over a 48-hour period. Two-way repeated ANOVA examining the effect of time period, *mdx* genotype and their potential interaction (A-H). ***p* < 0.01, *** *p* < 0.001, and *****p* < 0.0001 using a Sidak's post-hoc test, n = 5-6 per group. Student's t-test examined WT versus mdx (I-L), n = 5-6 per group. Values are represented as mean ± SEM.

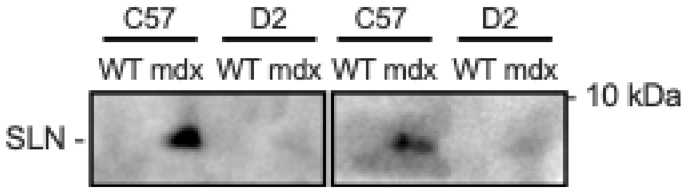

Supplementary Figure 3. Representative western blot image illustrating ectopic expression of SLN in gastrocnemius muscles from C57 and D2 *mdx* mice, whereas SLN could not be detected in WT muscle. Related to Figure 2.
